# Calcifying Epithelial Odontogenic Tumor: A Case Report

**DOI:** 10.31729/jnma.4842

**Published:** 2020-03-31

**Authors:** Radha Baral, Dipshikha Bajracharya, Bidhata Ojha, Gaurav Karna

**Affiliations:** 1 Department of Oral Pathology, Kantipur Dental College, Kathmandu, Nepal; 2 National Academy of Medical Science, Kathmandu, Nepal

**Keywords:** *amyloid*, *case report*, *mandible*, *odontogenic tumor*

## Abstract

Calcifying epithelial odontogenic tumor is a rare benign odontogenic tumor which accounts for approximately 1% of the entire odontogenic tumor. It was firstly described by Pindborg, and thus, is also referred to as the “Pindborg tumor”. Histologically, Pindborg tumor consists of three distinct histological compo nents: sheets of polyhedral epithelial cells, amyloid like deposits, and calcifications. This case report describes a case of Calcifying epithelial odontogenic tumor in 26 years old female patient presented with the swelling in right posterior region of mandible. Taking into account of the aggressive nature of the lesion segmental resection of the mandible followed by reconstruction was planned for treatment. This case report highlights the importance of appropriate clinical, radiographical and histological correlation for the correct diagnosis and treatment of Pindborg tumor.

## INTRODUCTION

Calcifying epithelial odontogenic tumor (CEOT) is a rare benign odontogenic tumor which accounts for approximately 1% of the entire odontogenic tumors. It was firstly described by Pindborg, and thus, is also referred to as the “Pindborg tumor”.^1^ It is an odontogenic neoplasm completely derived from epithelial tissue. Histogenesis of the tumor is still not clear. Some pathologists believe that CEOT is derived from the stratum intermedium layer of the enamel organ in the tooth development stage while others argue that this tumor may arise from remnants of the primitive dental lamina found in the initial stage of odontogenesis.^2^

## CASE REPORT

**Figure 1:**
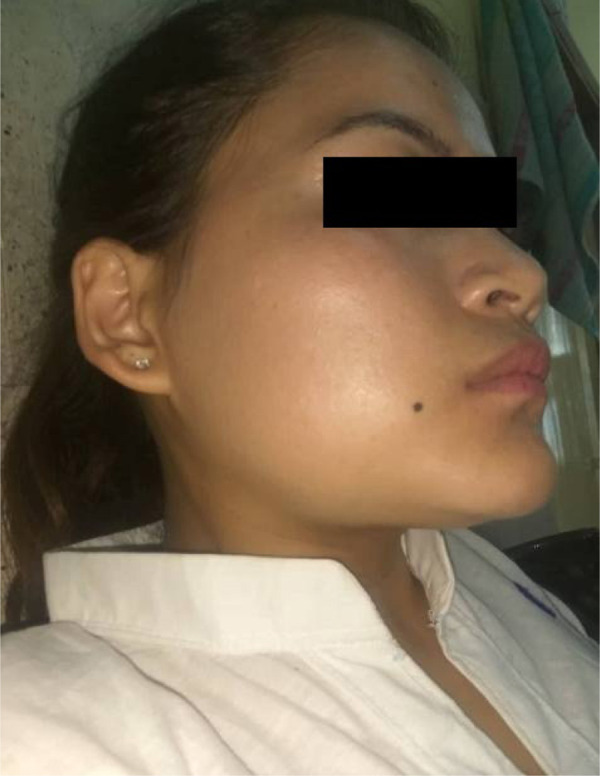
Extraoral swelling in the right lower back region.

**Figure 2:**
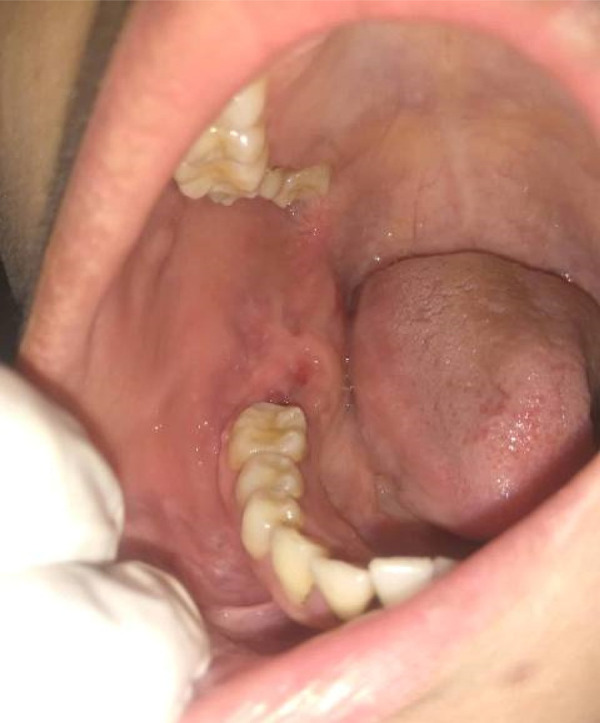
Intraoral picture one week after incisional biopsy along with the extraction of 47.

A 26 years old female patient presented with the chief complaint of swelling in the right lower back region of the jaw for 1.5 years. The swelling was associated with pain for the past 6 months. The pain was intermittent and dull aching in nature. The patient also complained of loosening of the teeth in the region of swelling. On extraoral examination swelling measuring approximately 3cm x 2cm in size was present which was causing mild facial asymmetry. The swelling was involving the right angle of the mandible and submandibular region and it was firm on palpation (Figure 1). The right submandibular lymph nodes were palpable but not tender. Intraoral examination of the lesion revealed swelling of about 5cm x 5cm in size in the right retromolar triangle region. The swelling was causing the obliteration of the buccal vestibule and was also involving the right mandibular second molar. The overlying mucosa appeared normal without ulceration (Figure 2). The swelling was firm on palpation and was involving 47 which was grade III mobile.

**Figure 3:**
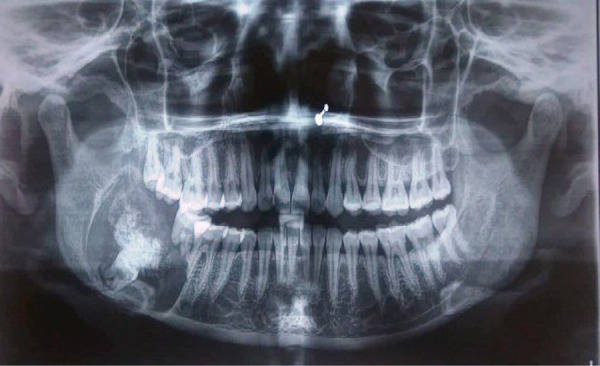
Orthopatomogram (OPG) showing mixed radiolucent and radiopaque lesion involving impacted mandibular right third molar.

A panoramic radiograph revealed mixed radiolucent and radiopaque lesion extending from the distal root of 46 anteriorly to ramus of mandible posteriorly. The lesion had distinct sclerotic border anteriorly and posteriorly with an indistinct border superiorly. Loss of continuity of the lower border of mandible could be noted inferiorly. The presence of a tooth-shaped radiopacity in conjunction with the inferior margin of the lesion suggestive of impacted 48 could be appreciated. The associated tooth 47 was displaced occlusally and both of its roots were resorbed. The lesion was also causing resorption of the distal root of the 46 (Figure 3).

Based on the clinical and radiographic findings, a differential diagnosis of Calcifying odontogenic cyst (COC) and a Dentigerous cyst was given. An incisional biopsy of the lesion was performed along with the extraction of 47.

**Figure 4:**
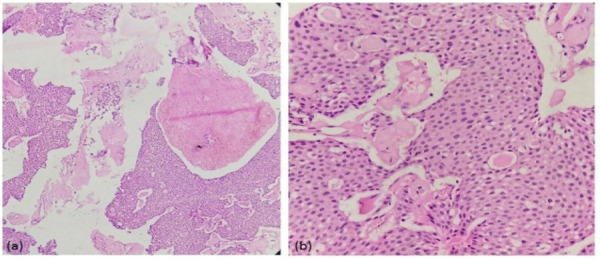
Photomicrograph showing sheets of closely packed epithelial cells with homogenous eosinophilic material (a) H & E 10X (b) H & E 40X.

On gross examination, multiple pieces of soft to firm tissue whitish in color together measuring 1.5cm x 1.5cm were received. Histopathological examination revealed tumor mass composed of closely packed sheets of polyhedral cells. The tumor cells had a well-outlined border with eosinophilic cytoplasm and central round nucleus. The presence of homogenous eosinophilic material could be appreciated within and between sheets of tumor cells. Small areas of basophilic calcification could also be noted (Figure 4a and 4b). The diagnosis of the dentigerous cyst was excluded in the absence of cystic nature and reduced enamel epithelium like lining. The presence of polyhedral cells instead of ameloblastomatous epithelium and ghost cells differentiated it histologically from COC.

Based upon these histological findings a final diagnosis of Calcifying epithelial odontogenic tumor (CEOT) was given. Segmental resection of the mandible followed by reconstruction was planned for treatment.

## DISCUSSION

CEOT is a rare benign odontogenic tumor which accounts for approximately 1% of all the odontogenic tumors.^3,4^ CEOTs are intraosseous comprising 96% of cases and approximately 6% of CEOTs are extraosseous. They tend to occur over a wide age range but usually predominate in the 3rd to 6th decades of life with almost equal sex predilection.^4^ Intraosseous tumors occur more often in the mandible than in the maxilla especially in the premolar and molar regions. Around 52% of these tumors are associated with impacted or unerupted teeth or odontomas, most commonly mandibular molars.^1,5^ The lesion was seen on the posterior mandible and was associated with impacted third molar in the present case.

Radiographically, the lesion usually consists of a radiolucent area, which may be well or poorly defined, uni or multilocular, containing radiopaque masses of varying size and opacity. When an unerupted tooth is associated with the tumor, the radiopacities tend to be located close to the tooth crown.^5,6^ In our case lesion had mixed radiopacity and radiolucency with well-defined borders. The lesion was associated with impacted third molar and radiopacity was in close association with the crown of the impacted tooth (Figure 3). Although CEOT is a benign tumor, it has variable biologic behavior ranging from mild to moderate invasiveness. The tumor grows by infiltration and produces cortical expansion and root resorption.^7^ In the present case, the tumor was aggressive and was causing cortical expansion and significant root resorption of the associated teeth.

Histopathologically, CEOT is composed of polyhedral neoplastic cells, which have abundant eosinophilic finely granular cytoplasm with nuclear pleomorphism and prominent nucleoli. Most of the cells are arranged in anastomosing sheet-like masses. An extracellular eosinophilic homogenous material staining like amyloid is characteristic of this tumor with concentric calcific deposits called Liesgang ring.^8,9^ Similar histopathological features were seen in the case presented here along with small areas of calcification (Figure 4a and 4b), however, nuclear pleomorphism was not significant in the tumor cells.

The treatment for CEOT has ranged from simple enucleation or curettage to radical and extensive resection such as hemi mandibulectomy or hemimaxillectomy.^2^ The choice should be individualized for each lesion because the radiological and histological features may differ from one lesion to another. Since the case reported was of great extension and rapid evolution, the treatment was right mandible segmental resection followed by reconstruction.

CEOT is an odontogenic tumor having distinct histology however, proper clinical, radiographical and histological correlation is required to arrive at correct diagnosis and treatment planning of this tumor.
